# Infodemics in an Era of Pandemics

**DOI:** 10.3389/ijph.2022.1605209

**Published:** 2022-08-17

**Authors:** Nina Emery, Adeline Dugerdil, Antoine Flahault

**Affiliations:** ^1^ Institute of Global Health, Faculty of Medicine, University of Geneva, Geneva, Switzerland; ^2^ School of Medicine, Faculty of Biology and Medicine, University of Lausanne, Lausanne, Switzerland

**Keywords:** monkeypox, pandemic, infodemic, misinformation, SARS CoV-2

What is monkeypox exactly and where does it come from? Could it be a side effect of SARS-CoV-2 vaccines? An intentionally released virus created by vaccines manufacturers? Is it a “gay disease?” Along with the number of monkeypox cases in non-endemic countries, misinformation and disinformation about them started multiplying on the internet. Differing in their intentionality, misinformation defines a “misleading or incorrect information” while disinformation is a “false information deliberately spread […] in order to influence public opinion […]” [1]. In their review, Ennab and Nawaz (2022) unravel some of the most common myths on the current outbreak [2]. While often absurd, old and recent events have taught us that the effect of misinformation must not be ignored.

In the early 1980s, the misbelief that HIV/AIDS only affected males having sex with males (MSM) delayed detection of other risk groups and triggered stigmatization of the gay community. Years of effort were (and are still) necessary to tackle this stigmatization and its many negative impacts on early diagnosis, treatment adherence and mental health, to name a few [3]. The overrepresentation of patients identifying as MSM in the recent monkeypox surge has also led to the misconception that it would be a “gay disease,” even though previous and recent cases diagnosed in females and children show that anyone is susceptible to get infected [4, 5]. One risk behind this misbelief, both among health care workers and the public, is to favor a “tunnel vision” focused on MSM that would hamper the broader detection of cases unrelated to this population. Nevertheless, it is still critical to effectively target risk groups with quality and unprejudiced information. As highlighted in the fight against HIV/AIDS, the actors most qualified to distribute such a message are those directly involved in the LGBTQI+ communities, such as Non-Governmental Organizations (NGOs) and activists, who have the necessary expertise and sensitivities to shape the most befitting message.

During the SARS-CoV-2 pandemic, the spread of mis- and dis-information has reached new heights. Its overabundance has been shown to reduce the efficiency of public health efforts to mitigate the SARS-CoV-2 dissemination, diverting people from positive health behavior and sometimes even making them adopt dangerous ones [6]. For instance, misinformation was shown to reduce intent to vaccinate against SARS-CoV-2 by more than 6 percentage points in the United Kingdom and the United States of America [6]. Indeed, there are strong links between SARS-CoV-2 and monkeypox disinformation. The majority of false claims on monkeypox debunked by the digital verification service of Agence France Presse (AFP) were related to SARS-CoV-2 vaccines [7]. Such false claims demonstrate that media manipulators used monkeypox as a vector to spread disinformation on SARS-CoV-2 vaccines safety, to benefit from the high number of web searches and reach wider audience.

As pointed out by Ennab and Nawaz (2022) and according to the WHO Information Network for Epidemics (EPI-WIN) [8], public health actors must strive to spread evidence-based and easily understandable information to all population groups, to counter misinformation. Indeed, public information campaigns have proven to be very effective: a retrospective analysis of non-pharmaceutical interventions to mitigate the spread of SARS-CoV-2 have ranked public information campaigns as the most efficient intervention, outperforming facial covering requirements, school closing and contact tracing [9]. Interestingly, it showed an even greater efficiency during the second wave of SARS-CoV-2 compared to the first one. According the authors, one potential reason for this increase could be an improved dissemination of information to the public across time.

If the fight against SARS-CoV-2 has led to more efficient communication channels with the population, this represents another reason to use them in the fight against monkeypox. However, it can be difficult to inform populations when there is still relatively little known about this disease. Although first identified in a human in 1970, the disease has been neglected since then despite an ever-increasing epidemiology and the multiplication of alarm signals [10]. A lot is still to be discovered and it is highly probable that some of the existing knowledge on monkeypox will be dismissed as research progresses. Thus, the necessity arises to be transparent on the very evolutive nature of research and to communicate uncertainties to the public as recommended by the WHO EPI-WIN [8]. Being over-assertive might result in a loss of trust to health authorities if evidence happens to change over time.

In conclusion, lessons from past outbreaks of emerging infectious diseases show that mis- and dis-information can be dangerously damaging. Thus, action must be taken urgently to spread evidence-based information on monkeypox, both in general population via public campaigns and among MSM community with the help of NGOs. While doing so, it is as important to communicate on what is known as what is unclear yet, in order to maintain trust.
